# Intensive Care Unit Nurses' Knowledge, Attitudes, and Practices Regarding Mechanical Ventilator Weaning and Associated Factors in Resource‐Limited Public Hospitals in Addis Ababa, Ethiopia

**DOI:** 10.1002/nop2.70759

**Published:** 2026-08-18

**Authors:** Ayto Addisu Negash, Dagmawi Anteneh Teferi, Mulugeta Tesfaye, Mehreteab Tadesse Woudineh, Tekiy Markos Bedore, Yitayew Ewnetu Mohammed, Maramawit Solomon, Adey Abebaw Bogale, Etsegenet Dege Dires, Dirijit Mamo

**Affiliations:** ^1^ Department of Emergency Medicine and Critical Care Worabe Comprehensive Specialized Hospital, Werabe University Werabe Ethiopia; ^2^ Department of Emergency Medicine and Critical Care Nigist Eleni Mohamed Memorial Comprehensive Specialized Hospital, Wachemo University Hosanna Ethiopia; ^3^ Department of Surgery St. Paul's Hospital Millennium Medical College Addis Ababa Ethiopia; ^4^ Department of Nursing St. Paul's Hospital Millennium Medical College Addis Ababa Ethiopia; ^5^ Department of Paediatric and Child Health St. Paul's Hospital Millennium Medical College Addis Ababa Ethiopia; ^6^ Department of Internal Medicine Worabe Comprehensive Specialized Hospital, Werabe University Werabe Ethiopia; ^7^ Department of Emergency Medicine and Critical Care St. Paul's Hospital Millennium Medical College Addis Ababa Ethiopia; ^8^ Department of Family Medicine St. Paul's Hospital Millennium Medical College Addis Ababa Ethiopia

**Keywords:** Ethiopia, geographic novelty, ICU nurses, mechanical ventilator weaning, resource‐limited settings

## Abstract

**Aim:**

To assess intensive care unit (ICU) nurses' knowledge, attitudes, and practices regarding mechanical ventilator weaning and associated factors in public hospitals in Addis Ababa, Ethiopia.

**Design:**

A hospital‐based cross‐sectional study.

**Methods:**

A cross‐sectional study was conducted among 275 ICU nurses from 11 public hospitals in Addis Ababa between April and October 2024. Data were collected using a structured self‐administered questionnaire. Descriptive statistics and multivariable logistic regression analyses were performed to identify factors associated with knowledge, attitudes, and practices. Statistical significance was set at *p* < 0.05.

**Data Sources:**

Primary data were collected using a structured questionnaire.

**Results:**

Good knowledge, positive attitudes, and good practices were reported by 56.7%, 94.9%, and 60.7% of nurses, respectively. Higher educational level was associated with positive attitudes and good practices, while more than 2 years of ICU experience was associated with good practice. Nurses aged 31–40 years were less likely to demonstrate good practice.

**Conclusion:**

Although most ICU nurses demonstrated positive attitudes toward mechanical ventilator weaning, important knowledge and practice gaps remain. Educational level and ICU experience were associated with ventilator weaning practices. Strengthening nursing education and standardized evidence‐based protocols may enhance critical care nursing practice.

**Implications for the Profession and/or Patient Care:**

Nurse‐focused continuing education, competency‐based training, and standardized ventilator weaning protocols may strengthen evidence‐based nursing practice and support safe patient care in resource‐limited ICUs.

**Impact:**

This study addresses limited evidence on ICU nurses' ventilator weaning competencies in resource‐limited settings. The findings identify knowledge and practice gaps and associated professional factors. The results may inform nursing education, clinical practice, and policy development in critical care settings.

**Reporting Method:**

Reported according to the STROBE guideline for cross‐sectional studies.

**Patient or Public Contribution:**

No patient or public contribution.

## 1 Introduction

### 1.1 Background

Mechanical ventilation (MV) is a critical therapeutic intervention frequently used for severely ill or injured patients in intensive care units (ICUs) (Hickey et al. 2024). Globally, up to 90% of ICU patients require mechanical ventilation for some portion of their stay (Perrie et al. 2014; HadiAtiyah et al. 2016; Botha 2012). For critically ill adults and children, MV serves as a life‐support system that maintains ventilation and oxygenation (Navalesi et al. 2014; Khalil et al. 2012), with most patients requiring short‐term support, while some may need prolonged assistance (Beduneau et al. 2017). In the United States, approximately 800,000 hospitalized patients, excluding neonates, require mechanical ventilation annually (Dotson 2013). Transitioning intubated patients to spontaneous breathing, either gradually or suddenly, is called ventilator weaning (Scalise and Votto 2005; Pradhan and Shrestha 2017; Boles et al. 2007). Successful weaning requires assessment of multiple clinical parameters, including hemodynamic stability, respiratory effort, cough and gag reflexes, absence of excessive secretions, mental status, metabolic stability, oxygenation, and minimal sedation (Dotson 2013; Boles et al. 2007). Proper weaning reduces re‐intubation, shortens hospital stay, lowers the risk of ventilator‐associated complications, and improves survival (Hickey et al. 2024).

### 1.2 Rationale

Effective weaning depends on nurses' knowledge, practical skills, and attitudes, with evidence‐based protocols such as checklists and guidelines helping standardize care (Boles et al. 2007; Hassen et al. 2023). Positive attitudes toward ventilator weaning, which include affective, behavioural, and cognitive components, influence nursing behaviour and decision‐making (Mullai Baalaaji 2021). Knowledge and attitudes are closely linked, as knowledge shapes behaviour and subsequent attitudes (Mullai Baalaaji 2021). Assessing ICU nurses' knowledge, attitudes, and practices (KAP) is essential to optimize education, enhance protocol‐directed care, and improve patient safety (Shehab et al. 2018). Gaps in knowledge or negative attitudes can delay weaning, increase hospital costs, and predispose patients to ventilator‐related complications (Mullai Baalaaji 2021; Shehab et al. 2018). Nurses have an ethical responsibility to practice safely, and evaluating KAP before implementing educational interventions ensures targeted and effective improvements (Shehab et al. 2018; Borkowska et al. 2015).

### 1.3 Research Question

Mechanical ventilator weaning is a critical component of intensive care nursing practice and directly influences patient outcomes, ICU length of stay, and healthcare costs. However, evidence on nurses' knowledge, attitudes, and practices regarding ventilator weaning remains limited in low‐ and middle‐income countries, where constraints in staffing, training opportunities, and resources often impact the quality of critical care. In Ethiopia, particularly in Addis Ababa, public hospitals manage a substantial proportion of critically ill patients, yet no recent study has systematically assessed ICU nurses' competence in ventilator weaning. Despite the central role of ICU nurses in assessing readiness for ventilator weaning and implementing evidence‐based weaning strategies, there is limited context‐specific evidence describing their knowledge, attitudes, and practices in Ethiopian public hospitals. Addressing this research gap is essential to identify modifiable nursing factors that can inform targeted educational interventions, standardized weaning protocols, and nursing policies aimed at improving the quality and safety of ventilator weaning in resource‐limited ICUs. This study aims to comprehensively evaluate the knowledge, attitudes, and practices of ICU nurses regarding ventilator weaning. Specifically, it quantifies nurses' knowledge of evidence‐based weaning protocols, examines their attitudes, explores clinical practices, including adherence to protocols, and investigates associations between nurses' demographic and professional characteristics and their knowledge, attitudes, and practices.

### 1.4 Significance/Expected Impact

By identifying gaps in knowledge, attitudes, or practices, this study provides actionable insights for the development of targeted educational programs and locally adapted protocols. The findings aim to enhance ICU weaning practices, improve patient outcomes, and inform policy development in resource‐limited ICU settings in Ethiopia and other low‐to middle‐income countries.

### 1.5 Objectives

#### 1.5.1 General Objectives

To assess intensive care unit (ICU) nurses' knowledge, attitudes, and practices regarding mechanical ventilator weaning and associated factors in resource‐limited public hospitals in Addis Ababa, Ethiopia.

#### 1.5.2 Specific Objectives

To assess ICU nurses' knowledge regarding mechanical ventilator weaning in resource‐limited public hospitals in Addis Ababa, Ethiopia.

To assess ICU nurses' attitudes toward mechanical ventilator weaning in resource‐limited public hospitals in Addis Ababa, Ethiopia.

To assess ICU nurses' practices related to mechanical ventilator weaning in resource‐limited public hospitals in Addis Ababa, Ethiopia.

To identify factors associated with ICU nurses' knowledge, attitudes, and practices regarding mechanical ventilator weaning.

## 2 Materials and Methods

### 2.1 Study Design

A cross‐sectional study was conducted among adult ICU nurses in public hospitals using a structured, self‐administered questionnaire to assess knowledge, attitudes, and practices related to mechanical ventilator weaning. This study is reported in accordance with the Strengthening the Reporting of Observational Studies in Epidemiology (STROBE) guidelines (von Elm et al. 2007). A completed STROBE checklist is provided as File S1.

### 2.2 Study Area

The study was conducted in 11 public hospitals with adult ICUs in Addis Ababa, the capital city of Ethiopia, and the seat of the African Union, with an estimated population of over 3.4 million.

### 2.3 Study Period

Data were collected from April 1 to October 1, 2024 G.C.

### 2.4 Population and Sampling Technique

The source population included all nurses employed in public hospitals in Addis Ababa, while the study population comprised nurses working in adult ICUs of the selected public hospitals. A census sampling approach was employed, and all ICU nurses who met the eligibility criteria were invited to participate.

### 2.5 Eligibility Criteria

The inclusion criteria for the study were nurses who were actively working in adult ICUs during the study period. Nurses who were on leave or not part of the regular ICU staff were excluded from participation.

### 2.6 Study Variables

The primary dependent variables in this study were nurses' knowledge, attitudes, and practices regarding mechanical ventilator weaning. Independent variables included demographic and professional characteristics such as age, sex, educational level, professional training, and years of ICU experience.

### 2.7 Data Collection Tools and Procedure

Data were collected using a structured, self‐administered questionnaire adapted from previously validated tools (Mullai Baalaaji 2021; Shehab et al. 2018; Borkowska et al. 2015) and modified to suit the local context following expert review. During the adaptation process, selected items related to advanced mechanical ventilation management, pulmonary function assessment, and arterial blood gas (ABG) interpretation were modified or excluded because these practices are not routinely available or consistently performed in many resource‐limited ICU settings in Ethiopia. The adapted questionnaire therefore focused on essential knowledge, attitudes, and practices related to ventilator weaning that are applicable to the available clinical resources while maintaining the original objective of assessing ICU nurses' competencies. The questionnaire was pretested on a small group of ICU nurses (excluded from the final analysis) to ensure clarity, reliability, and content validity.

The questionnaire consisted of four sections. Section A assessed socio‐demographic characteristics and previous training exposure using six items with categorical response options. Section B assessed ICU nurses' knowledge regarding mechanical ventilator weaning using 15 items, including multiple‐choice questions, yes/no questions, Likert‐scale self‐rating questions, and clinical scenario‐based questions related to ventilator management, arterial blood gas interpretation, and weaning decision‐making. Section C assessed attitudes toward mechanical ventilator weaning using 12 items, including Likert‐scale questions ranging from strongly disagree to strongly agree and 1–10 rating scales. Section D assessed nurses' practices related to ventilator weaning using 13 items, including yes/no responses and frequency‐based Likert responses (never, sometimes, almost always, and always).

The scoring and categorization of knowledge, attitude, and practice were based on a modified Bloom's cut‐off approach. Knowledge scores were calculated from the relevant knowledge items, and scores ≥ 60% were classified as good knowledge, while scores < 60% were classified as poor knowledge. Practice scores were similarly categorized, with scores ≥ 60% indicating good practice and scores < 60% indicating poor practice. Attitude scores were categorized using a higher threshold, with scores ≥ 80% considered a positive attitude and scores < 80% considered a negative attitude.

The 60% cut‐off for knowledge and practice was selected based on the modified Bloom's classification approach and considering the contextual variation in ICU nursing practice, particularly in resource‐limited settings. ICU nurses working in such environments may face challenges related to limited access to standardized weaning protocols, updated clinical guidelines, advanced monitoring technologies, and specialized training opportunities. Therefore, this threshold was considered appropriate to identify acceptable levels of knowledge and practice while acknowledging variations in available resources and clinical exposure. The 80% cut‐off for attitude was selected to represent a stronger level of professional agreement toward evidence‐based weaning practices. Data collectors received training to standardize the administration of the questionnaire across all hospitals. Questionnaires were completed voluntarily and anonymously to minimize social desirability bias and encourage honest responses.

### 2.8 Operational Definitions

*Knowledge:* Refers to nurses' understanding of mechanical ventilator weaning, assessed via a structured questionnaire. Scores ≥ 60% were classified as good knowledge, and < 60% as poor.

*Attitude:* Refers to nurses' perspective toward ventilator weaning, measured using a structured attitude questionnaire. Scores ≥ 80% were classified as positive, and < 80% as negative.

*Practice:* Refers to the nurses' ability to manage mechanically ventilated patients, including application of knowledge and skills. Scores ≥ 60% indicated good practice, and < 60% indicated poor practice.

*Training exposure:* Training on the weaning protocol and daily assessment was operationally defined as any formal educational exposure received by ICU nurses from their institution or other recognized sources that addressed mechanical ventilator weaning principles, protocol application, or daily assessment of patients undergoing weaning. Training status was assessed as a dichotomous variable (Yes/No).

### 2.9 Sample Size

A total of 275 ICU nurses participated from 303 eligible nurses, resulting in a response rate of 90.8%. As a census approach was used, a formal sample size calculation was not required.

### 2.10 Statistical Analysis and Data Management

Completed questionnaires were checked for completeness before data entry into Epi Data Manager version 4.4.2. Data were subsequently analysed using SPSS version 27.

Descriptive statistics, including frequencies and percentages, were calculated to summarize the data. Associations between categorical variables were examined using chi‐square tests, while logistic regression analysis was applied to evaluate relationships between independent and dependent variables. Findings were presented using frequencies, percentages, tables, and narrative summaries. Bivariable logistic regression analyses were initially performed to examine the association between each independent variable and the outcome variables (knowledge, attitude, and practice). Variables with a *p*‐value < 0.25 in the bivariable analysis, along with variables considered clinically relevant based on previous literature and the study objectives, were entered into the multivariable logistic regression models. Adjusted odds ratios (AORs) with 95% confidence intervals (CIs) were calculated, and variables with a *p*‐value < 0.05 were considered statistically significant.

Prior to fitting the multivariable logistic regression models, the assumptions of the analysis were assessed. Multicollinearity among independent variables was evaluated using the variance inflation factor (VIF) and tolerance statistics. A VIF value < 5 and tolerance value > 0.1 were considered acceptable, indicating no significant multicollinearity among the predictor variables. Model fitness was assessed before interpretation of the regression results.

Data completeness was assessed during data cleaning and management. All questionnaires were reviewed for completeness, and no substantial missing data were identified; therefore, missing data imputation was not required.

### 2.11 Ethical Declaration

*Ethical Approval and Consent to Participate:* This study was approved by the Institutional Review Board of St. Paul's Hospital Millennium Medical College (SPHMMC) Institutional Review Board, Approval No IRB/2024/150, and was conducted in accordance with the Declaration of Helsinki. Written informed consent was obtained from all participating nurses prior to enrolment. Participants were fully informed about the study's purpose, procedures, potential risks and benefits, and their right to withdraw at any time without consequence. All responses were anonymized to ensure confidentiality.

## 3 Result

### 3.1 Socio‐Demographic Characteristics

Out of 303 eligible nurses, 275 participated in the study, yielding a high response rate of 90.7%. The sample was almost evenly distributed by sex, with 137 (49.8%) males and 138 (50.2%) females. Regarding educational status, 199 (72.4%) held a basic nursing degree, 61 (22.2%) had a Master's degree, and 15 (5.5%) had a diploma. More than half of the respondents, 144 (52.4%) had more than 2 years of ICU experience, while 87 (31.6%) had 6 months to 2 years and 44 (16.0%) less than 6 months of experience. In terms of training, 163 (59.3%) had received general training on daily assessments, but only 92 (33.5%) and 85 (30.9%) had training on weaning protocols and guidelines, respectively (Table 1).

**TABLE 1:** Socio‐demographic characteristics of adult ICU nurses, 2024 (*N* = 275).

Variable	Category	Frequency (*n*)	Percentage (%)
Sex			
	Male	137	49.8
	Female	138	50.2
Age			
	< 30	157	57.1
	31–40	112	40.7
	41–50	6	2.2
Marital status			
	Married	112	40.7
	Single	162	58.9
Educational status			
	Diploma	15	5.5
	BSc	199	72.4
	Master's	61	22.2
Work experience			
	< 6 months	44	16.0
	6 months–2 years	87	31.6
	> 2 years	144	52.4
Training on daily assessment			
	Yes	112	40.7
	No	163	59.3
Training on weaning protocol			
	Yes	92	33.5
	No	183	66.5
Training on weaning guideline			
	Yes	85	30.9
	No	190	69.1

### 3.2 Knowledge of ICU Nurses Regarding Mechanical Ventilator Weaning

Among the ICU nurses surveyed, 156 (56.7%) had good knowledge regarding mechanical ventilator weaning, whereas 119 (43.3%) exhibited poor knowledge. When asked to self‐assess, 140 (50.9%) rated their knowledge as excellent, 97 (35.3%) as good, and 38 (13.8%) as poor. Specific to the weaning protocol, 123 (44.7%) considered their knowledge excellent, 120 (43.6%) as good, and 32 (11.6%) as poor.

Analysis of specific competencies revealed notable gaps. Only 74 (26.9%) of nurses could correctly identify how to improve PaO_2_, and 87 (31.6%) knew the correct method to correct PaCO_2_. Knowledge of normal respiratory rate during weaning was high, with 205 (74.5%) answering correctly. However, 113 (41.1%) were unable to identify cardiovascular signs indicating weaning intolerance. Regarding spontaneous breathing trials (SBTs), 167 (60.7%) correctly identified continuous positive airway pressure (CPAP) as an appropriate method. Case‐based questions highlighted further deficiencies, with 121 (44.0%) unable to answer patient scenarios correctly. Hospital‐level analysis showed variation in knowledge. Ras Desta Hospital had the highest proportion of nurses with good knowledge, at 13 (68.4%), followed by SPHMMC with 30 (68.2%), and TASH with 17 (63.0%) (Table 2).

**TABLE 2:** All public hospitals' knowledge proportion, Addis Ababa, Ethiopia, 2024.

Variable	Hospital	Good knowledge *n* (%)	Poor knowledge *n* (%)
Hospital			
	Zewditu	5 (23.8)	16 (76.2)
	Menelik	6 (37.5)	10 (62.5)
	Yekatit 12	11 (57.9)	8 (42.1)
	AaBET	23 (52.3)	21 (47.7)
	ALERT	15 (57.7)	11 (42.3)
	TASH	17 (63.0)	10 (37.0)
	SPHMMC	30 (68.2)	14 (31.8)
	EKA	15 (60.0)	10 (40.0)
	St. Peter	11 (61.1)	7 (38.9)
	Tirunesh Beijing	10 (62.5)	6 (37.5)
	Ras Desta	13 (68.4)	6 (31.6)

Multivariable logistic regression analysis indicated that none of the assessed variables, including age, sex, educational level, training, or availability of protocols, were statistically significant predictors of good knowledge regarding mechanical ventilator weaning. Nurses with more than 2 years of ICU experience were 1.31 times more likely to demonstrate good knowledge compared to those with less than 6 months' experience (AOR = 1.31, 95% CI: 0.83–2.30), although this association was not statistically significant.

### 3.3 Attitude of ICU Nurses Regarding Mechanical Ventilator Weaning

Among the ICU nurses surveyed, 261 (94.9%) had a positive attitude toward weaning patients from mechanical ventilation. Specifically, 161 (58.5%) agreed that weaning is part of nursing care, and 63 (22.9%) strongly agreed with this statement. Over half of the nurses, 152 (55.3%), believed that following a weaning protocol increased their professional autonomy, while 49.5% agreed that protocols provided structured guidance for practice. Furthermore, 110 (40.0%) perceived the use of protocols as beneficial for patient outcomes.

Factors associated with nurses' attitudes were examined using bi‐variable and multivariable logistic regression analyses. Female nurses constituted 61.5% of those with a poor attitude and 49.6% of those with a good attitude; however, after adjustment, there was no statistically significant difference between females and males (AOR = 0.61, 95% CI: 0.196–1.931, *p* = 0.4). Age also did not show a significant association with attitude, with nurses aged 31–40 years having an AOR of 1.64 (95% CI: 0.493–5.471, *p* = 0.41) compared to those under 30 years. Similarly, work experience was not significantly associated with attitude after adjustment.

Educational status was strongly associated with ICU nurses' attitudes. Nurses holding a BSc degree were 15.44 times more likely to have a good attitude compared to those with a basic nursing diploma (AOR = 15.44, 95% CI: 3.918–60.844, *p* < 0.001). Nurses with a Master's degree also demonstrated higher odds of a positive attitude (AOR = 14.98, 95% CI: 2.485–90.329, *p* = 0.003). Training on the weaning protocol or guideline did not significantly predict attitude in the multivariable analysis, although the absence of training on the guideline showed a trend toward poorer attitude (AOR = 2.75, 95% CI: 0.896–8.453, *p* = 0.07) (Table 3).

**TABLE 3:** Factors associated with ICU nurses' attitude on mechanical ventilator weaning, Addis Ababa, Ethiopia, 2024 (*N* = 275).

Variable	Category	Poor *n* (%)	Good *n* (%)	COR (95% CI)	*p*	AOR (95% CI)
Sex						
	Male	5 (38.5)	132 (50.4)	1	—	—
	Female	8 (61.5)	130 (49.6)	0.6	0.4	0.61 (0.196–1.931)
Age						
	< 30	9 (69.2)	148 (56.5)	1	—	—
	31–40	4 (30.8)	108 (41.2)	1.6	0.41	1.64 (0.493–5.471)
	41–50	0 (0.0)	6 (2.3)	—	—	—
Work experience						
	< 6 months	3 (23.3)	41 (15.6)	1	—	—
	6 months–2 years	10 (76.9)	77 (29.4)	0.56	0.4	0.563 (0.147–2.162)
	> 2 years	0 (0.0)	144 (55.0)	—	0.9	—
Educational status						
	Basic diploma	5 (38.5)	10 (3.8)	1	—	—
	BSc	6 (46.2)	193 (73.7)	16.0	0.00*	15.44 (3.918–60.844)
	Master's	2 (15.4)	59 (22.5)	14.7	0.003*	14.98 (2.485–90.329)
Training on weaning protocol						
	Yes	5 (38.5)	87 (33.2)	1	—	—
	No	8 (61.5)	175 (66.8)	1.2	0.69	1.25 (0.399–3.957)
Training on weaning guideline						
	Yes	7 (53.8)	78 (29.8)	1	—	—
	No	6 (46.2)	184 (70.2)	2.7	0.07	2.75 (0.896–8.453)

*Note:* *p*‐values < 0.05 were considered statistically significant. The reference group (“1”) represents the baseline category for each variable.

### 3.4 Practice of ICU Nurses Regarding Mechanical Ventilator Weaning

Hospital‐level analysis revealed notable variation in ICU nurses' practice across public hospitals. Ras Desta Hospital had the highest proportion of nurses demonstrating good practice, with 16 (84.2%) nurses performing well, followed by SPHMMC with 33 (75%) and ALERT Hospital with 30 (61.5%). In contrast, TASH and Menelik Hospitals had lower proportions of good practice, with 11 (40.7%) and 7 (43.8%) respectively. Several hospitals, including St. Peter and Menelik, showed a higher proportion of nurses with poor practice than good practice (Table 4).

**TABLE 4:** Hospital‐level distribution of ICU nurses' practice on mechanical ventilator weaning (*N* = 275).

Variable	Hospital	Good practice *n* (%)	Poor practice *n* (%)
Hospital			
	Zewditu	13 (61.9)	8 (38.1)
	Menelik	7 (43.8)	9 (56.3)
	Yekatit 12	11 (57.9)	8 (42.1)
	AaBET	30 (68.2)	14 (31.8)
	ALERT	16 (61.5)	10 (38.5)
	TASH	11 (40.7)	16 (59.3)
	SPHMMC	33 (75.0)	11 (25.0)
	EKA	15 (60.0)	10 (40.0)
	St. Peter	8 (44.4)	10 (55.6)
	Tirunesh Beijing	7 (43.8)	9 (56.3)
	Ras Desta	16 (84.2)	3 (15.8)

Overall, 167 (60.7%) of ICU nurses reported good mechanical ventilator weaning practice, while 108 (39.3%) exhibited poor practice. Participation in daily weaning plans was inconsistent, with only 82 (29.8%) reporting regular involvement. Assessment of pulmonary functional parameters was limited, as only 32 (11.6%) consistently performed these evaluations, and 107 (38.9%) reliably assessed arterial blood gases (ABGs) prior to weaning. Most nurses, 203 (73.8%), appropriately documented patient care. The most commonly used weaning modes were T‐tube (57, 20.7%), SIMV (64, 23.3%), and CPAP (73, 26.5%).

Factors associated with practice were analysed using multivariable logistic regression. Female nurses had slightly higher odds of demonstrating good practice compared to males, although this was not statistically significant (AOR = 1.28, 95% CI: 0.489–3.346, *p* = 0.6). Age was a significant predictor: nurses aged 31–40 years were less likely to demonstrate good practice than those under 30 years (AOR = 0.138, 95% CI: 0.038–0.487, *p* = 0.002). Work experience was positively associated with practice, with nurses having more than 2 years of ICU experience showing significantly higher odds of good practice compared with those with less than 6 months of experience (AOR = 4.46, 95% CI: 1.71–28.07, *p* = 0.01).

Educational status was significantly associated with practice. Nurses holding a BSc degree were over seven times more likely to demonstrate good practice compared to diploma holders (AOR = 7.25, 95% CI: 1.497–35.140, *p* = 0.01), and those with a Master's degree were more than ten times as likely to perform well (AOR = 10.4, 95% CI: 1.458–74.22, *p* = 0.02). Training on the weaning protocol and guideline did not show statistically significant associations with practice in the multivariable analysis (Table 5).

**TABLE 5:** Factors associated with practice of ICU nurses on mechanical ventilator weaning (*N* = 275).

Variable	Category	Poor *n* (%)	Good *n* (%)	COR (95% CI)	*p*	AOR (95% CI)
Sex						
	Male	10 (9.3)	127 (76.0)	1	—	—
	Female	98 (91.7)	40 (24.0)	1.2	0.6	1.28 (0.489–3.346)
Age						
	< 30	4 (22.2)	153 (59.5)	1	—	—
	31–40	14 (77.8)	98 (38.1)	0.18	0.002	0.138 (0.038–0.487)
	41–50	0 (0.0)	6 (2.3)	—	—	—
Work experience						
	< 6 months	4 (2.2)	40 (15.6)	1	—	—
	6 months–2 years	12 (66.7)	75 (29.2)	0.62	0.23	0.44 (0.118–1.670)
	> 2 years	2 (11.2)	142 (55.3)	7.1	0.01	4.46 (1.71–28.07)
Educational status						
	Diploma	4 (22.2)	11 (4.3)	1	—	—
	BSc	11 (4.3)	188 (73.2)	6.2	0.01	7.25 (1.497–35.140)
	Master's	3 (16.7)	58 (22.6)	7.3	0.02	10.4 (1.458–74.22)
Training on weaning protocol						
	Yes	3 (16.7)	89 (34.6)	1	—	—
	No	15 (83.3)	168 (65.4)	0.37	0.13	1.04 (0.106–1.339)
Training on weaning guideline						
	Yes	5 (27.8)	80 (31.1)	1	—	—
	No	13 (72.2)	177 (68.9)	0.8	0.76	0.85 (0.293–2.468)

*Note:* Percentages are column percentages and indicate the distribution of characteristics within poor and good practice categories. The reference category for each variable is indicated as ‘1’.

**Summary Interpretation:** Among 275 ICU nurses, 156 (56.7%) had good knowledge of mechanical ventilator weaning based on the predefined cutoff score of ≥ 60% for good knowledge. Specific gaps included correcting PaO_2_ (26.9%) and PaCO_2_ (31.6%), although 74.5% correctly identified the normal respiratory rate. Attitudes were generally positive, with 161 (58.5%) agreeing that weaning is part of nursing care. Educational status was significantly associated with attitude, as nurses with a BSc or Master's degree were more likely to have a positive attitude (AOR = 15.44 and 14.98, respectively). Approximately three‐fifths of nurses demonstrated good practice. Age, work experience, and educational level were significant predictors of practice, while training and sex were not. Nurses with more than 2 years of ICU experience had higher odds of good practice (AOR = 4.46, 95% CI: 1.71–28.07), while nurses with BSc and Master's degrees demonstrated higher odds compared with diploma holders. Overall, the findings suggest opportunities to strengthen competency‐based education, standardized weaning protocols, and ongoing competency assessment.

## 4 Discussion

Mechanical ventilation (MV) is a critical life‐saving intervention for patients in intensive care units (ICUs). However, the process of weaning these patients off ventilation requires advanced clinical judgement, coordinated care, and skilled nursing practice. Our study evaluated the knowledge, attitudes, and practices (KAP) of ICU nurses regarding the weaning process for mechanical ventilators across eleven public hospitals in Addis Ababa, Ethiopia. The findings revealed significant gaps in formal training, the availability of protocols, and the consistent application of evidence‐based weaning practices.

In our sample of 275 ICU nurses, most were relatively young with moderate experience in the ICU. Nurse demographics can influence knowledge, attitudes, and practices, as less experienced nurses may have theoretical knowledge but limited practical exposure. Conversely, more experienced nurses might rely on established routines that do not align with updated protocols. Our findings showed that 183 (66.5%) of the nurses had not received formal training in ventilator weaning, and 190 (69.1%) reported that there were no standardized weaning protocols in their units. This highlights a significant gap in institutional support and professional development. These findings are consistent with previous research suggesting that inadequate formal education may limit adherence to evidence‐based ventilator weaning practices and contribute to poorer weaning outcomes (Fernando et al. 2019).

While 156 (56.7%) of nurses showed a good understanding of weaning practices, only 167 (60.7%) reported implementing these practices appropriately. Although adequate practice was reported by a slightly higher proportion of nurses than those demonstrating good knowledge, these domains were assessed independently using different questionnaire items and scoring criteria. In addition, practice was self‐reported and may have been influenced by recall or social desirability bias. Nurses may also perform recommended weaning practices by following established unit routines, institutional protocols, or multidisciplinary team decisions despite having gaps in their theoretical knowledge of mechanical ventilator weaning. Important deficiencies included pulmonary function assessment, ABG assessment, and knowledge regarding spontaneous breathing trials. Similar trends have been noted in previous studies, where inadequate adherence to protocols and insufficient training hindered nurse‐led weaning performance (Perkins et al. 2018).

Although evidence supports the effectiveness of structured nurse‐led weaning protocols in improving patient outcomes, our findings indicate that only 40% of nurses perceived weaning protocols as beneficial. Previous studies have demonstrated that protocol‐directed weaning reduces the duration of mechanical ventilation, ICU length of stay, ventilator‐associated complications, and improves patient safety without increasing extubation failure or mortality (Perkins et al. 2018; Tonnelier et al. 2005). Taken together with previous evidence, our findings support consideration of standardized protocols and competency‐based education.

Education and training are crucial for enhancing nurse competence and improving patient outcomes. A recent scoping review indicated that education on mechanical ventilator weaning significantly reduced the duration of mechanical ventilation, while also boosting nursing confidence and adherence to protocols (Kimura et al. 2023). This highlights that knowledge alone is insufficient; structured training programs and continuous professional development are essential for safe and effective practice.

In contrast to some previous studies, none of the variables examined, including age, sex, educational level, ICU work experience, training, or availability of weaning protocols, were independently associated with nurses' knowledge after adjustment for potential confounders. This finding may indicate that knowledge gaps were relatively widespread across the study population rather than being confined to specific demographic or professional groups. It may also reflect unmeasured factors, such as differences in undergraduate education, access to continuing professional development, individual learning opportunities, or routine exposure to mechanically ventilated patients, which were not assessed in the present study. Similar findings have been reported in studies where knowledge appeared to be influenced more by institutional and educational factors than by individual demographic characteristics (Fernando et al. 2019; Tonnelier et al. 2005).

Interestingly, formal training was not independently associated with knowledge, attitude, or practice in the multivariable analysis. This may reflect differences in the quality, duration, or content of training programs, the limited number of nurses who received weaning‐specific training, or inadequate reinforcement of knowledge in routine clinical practice (Tonnelier et al. 2005). In addition, training was measured only as a dichotomous exposure without evaluating its duration, quality, recency, or educational content, which may have reduced the ability to detect meaningful associations.

Our study found that age was significantly associated with weaning practice. Specifically, nurses aged 31–40 years had significantly lower odds of demonstrating good weaning practices than nurses younger than 30 years (AOR = 0.138, 95% CI: 0.038–0.487, *p* = 0.002).

Although the cross‐sectional design does not permit causal inference, this finding may reflect differences in workload, professional responsibilities, or other unmeasured workplace factors among nurses in this age group. In addition to age, longer ICU work experience was associated with better weaning practice. Nurses with more than 2 years of ICU experience had higher odds of demonstrating good practice compared with those with less than 6 months of experience. This finding may reflect the cumulative benefits of greater exposure to mechanically ventilated patients, increased familiarity with ICU routines and ventilator management, enhanced clinical assessment and decision‐making skills, and more active participation in multidisciplinary rounds. Nevertheless, the relatively wide confidence interval indicates some imprecision in the estimated effect and suggests that the finding should be interpreted cautiously. However, continued professional development remains essential because experience alone may not guarantee adherence to evidence‐based weaning protocols.

Educational attainment was also strongly associated with nurses' attitudes toward mechanical ventilator weaning. Nurses holding BSc and Master's degrees demonstrated substantially higher odds of a positive attitude than diploma‐prepared nurses. Higher educational preparation may provide greater exposure to evidence‐based practice, strengthen critical appraisal skills, and increase confidence in clinical decision‐making, thereby fostering more favourable attitudes toward protocolized ventilator weaning (Fernando et al. 2019; Tonnelier et al. 2005). However, these estimates should be interpreted cautiously because the wide confidence intervals indicate limited precision, probably reflecting the small number of diploma‐prepared nurses included in the study. Future studies with a larger and more balanced distribution of educational categories are needed to confirm these findings.

Educational attainment was also significantly associated with practice. Nurses with BSc and Master's‐level education demonstrated higher odds of good practice compared with diploma‐level nurses. Higher educational preparation may enhance critical thinking, evidence appraisal skills, and confidence in applying complex clinical interventions such as mechanical ventilator weaning. This finding suggests that higher educational preparation may facilitate the translation of evidence‐based knowledge into clinical practice; however, prospective studies are needed to clarify the mechanisms underlying this association (Perkins et al. 2018). The observed variation in knowledge and practice across participating hospitals also suggests that institutional factors, including local leadership, availability of protocols, staffing patterns, and opportunities for continuing professional development, may influence ventilator weaning practices.

**Implications for Practice:** The findings identify specific areas that may benefit from targeted quality improvement within participating ICUs. Knowledge gaps in arterial blood gas interpretation, management of oxygenation and ventilation parameters, and assessment of pulmonary function suggest the need for focused educational initiatives addressing these aspects of mechanical ventilator weaning. The low proportion of nurses reporting training on weaning protocols and guidelines, together with inconsistent participation in daily weaning plans and pre‐weaning patient assessments, indicates opportunities to strengthen the quality, consistency, and competency‐based delivery of ventilator weaning education.

The observed variation in knowledge and practice across hospitals also suggests that local institutional factors may influence mechanical ventilator weaning practices. Furthermore, the association of educational attainment with nurses' attitudes and practices highlights the importance of continuing professional development to strengthen competencies related to ventilator weaning. These findings may assist hospital administrators and ICU leaders in identifying priority areas for educational and quality improvement initiatives tailored to their local context. These findings also have important implications for nursing practice by demonstrating that strengthening ICU nurses' knowledge and competencies can support more consistent, evidence‐based ventilator weaning and enhance patient safety. From a scientific perspective, this study fills an important evidence gap by providing context‐specific data from Ethiopian public hospitals, which can inform future nursing education, intervention studies, and policy development for critical care nursing in resource‐limited settings.

**Limitations:** The cross‐sectional design and reliance on self‐reported data may introduce biases related to social desirability and recall. The lack of direct observation limits the ability to verify actual practices. Patient‐level outcomes, such as re‐intubation rates or length of stay in the ICU, were not assessed, which restricts our ability to link nurses' knowledge, attitudes, and practices (KAP) directly to clinical outcomes. Additionally, variations in staffing, case mix, and institutional policies across different ICUs may affect the generalizability of the results.

The study assessed only the presence or absence of training exposure and did not evaluate the content, duration, frequency, or delivery method of training. Therefore, variations in training quality and intensity that may influence nurses' knowledge, attitudes, and practices could not be assessed. Mechanical ventilator weaning is a multidisciplinary process requiring coordinated input from physicians, physiotherapists, respiratory therapists (where available), pharmacists, and ICU nurses. Although the present study focused on ICU nurses because of their central role in patient assessment and implementation of weaning interventions, collaboration among all members of the critical care team is essential for successful weaning and optimal patient outcomes. Although this study included ICU nurses from multiple public hospitals, clustering at the hospital level was not accounted for in the statistical analysis. This was because the majority of participating hospitals had similar tertiary‐level ICU characteristics, resulting in limited institutional‐level variability for robust multilevel modelling. Therefore, potential differences related to hospital‐level factors may not have been fully captured. In addition, practice measures were based on self‐reported responses, which may be affected by recall or social desirability bias. The findings should therefore be interpreted as associations rather than causal relationships.

**Recommendation:** Future studies should include a broader range of healthcare institutions with greater variation in hospital level, ICU organization, available resources, and service capacity. Such studies should consider applying multilevel analytical approaches to better understand the independent contributions of individual nurse characteristics and institutional factors on ICU nurses' knowledge, attitudes, and practices related to mechanical ventilator weaning. Future prospective or interventional studies should evaluate whether implementing standardized nurse‐led weaning protocols and competency‐based educational programs improves nurses' practices and patient outcomes.

## 5 Conclusion

Although most ICU nurses demonstrated positive attitudes toward mechanical ventilator weaning, important knowledge and practice gaps remain, with good knowledge and practice reported among 56.7% and 60.7% of nurses, respectively. Deficiencies were identified in key areas of ventilator weaning competency, including formal training, protocol availability and implementation, and consistent use of evidence‐based practices. Educational level and ICU experience were significant factors associated with ventilator weaning practices. These findings underscore the importance of strengthening nurse‐focused continuing education, competency‐based training, and standardized evidence‐based ventilator weaning protocols to enhance critical care nursing practice, improve clinical decision‐making, and support safe, high‐quality patient care in resource‐limited ICU settings.

## Author Contributions

**Ayto Addisu Negash:** conceptualization, investigation, funding acquisition, writing – original draft, methodology, validation, visualization, writing – review and editing, software, formal analysis, project administration, data curation, supervision, and resources. **Dagmawi Anteneh Teferi:** methodology, validation, writing – review and editing, supervision. **Mulugeta Tesfaye:** methodology, validation, supervision, writing – review and editing. **Mehreteab Tadesse Woudineh:** methodology, validation, writing – review and editing, supervision. **Tekiy Markos Bedore:** validation, formal analysis, writing – review and editing. **Yitayew Ewnetu Mohammed:** validation, writing – review and editing, formal analysis. **Maramawit Solomon:** validation, writing – review and editing, formal analysis. **Adey Abebaw Bogale:** validation, writing – review and editing, formal analysis. **Etsegenet Dege Dires:** validation, writing – review and editing, formal analysis. **Dirijit Mamo:** validation, writing – review and editing, formal analysis.

## Funding

The authors have nothing to report.

## Conflicts of Interest

The authors declare no conflicts of interest.

## Supporting information


**Data S1:** STROBE Statement—checklist of items that should be included in reports of observational studies

## Data Availability

The datasets generated and/or analysed during the current study are not publicly available due to ethical and confidentiality considerations but are available from the corresponding author on reasonable request.
